# Analysis of prevalence and prognosis of type 2 diabetes mellitus in patients with acute exacerbation of COPD

**DOI:** 10.1186/s12890-020-01371-9

**Published:** 2021-01-06

**Authors:** Li Lin, Jianxin Shi, Jian Kang, Qiuyue Wang

**Affiliations:** Department of Pulmonary and Critical Care Medicine, Institute of Respiratory Disease, The First Hospital of China Medical University, No. 155 Nanjing Street, North, Shenyang, 110001 China

**Keywords:** COPD, Acute exacerbation of COPD, Diabetes mellitus, Coronary heart disease, Hypertension, Short-term prognosis

## Abstract

**Background:**

For patients with acute exacerbation of COPD (AECOPD), type 2 diabetes mellitus (T2DM) as comorbidity have poor outcomes. However, data on the impact of previously diagnosed and new- diagnosed T2DM in such a patient population is lacking.

**Methods:**

Inpatients diagnosed with AECOPD in the department of Pulmonary and Critical Care Medicine of The First Hospital of China Medical University during 2011–2017 were enrolled. Data on demography, prevalence of type 2 DM, other comorbidities, hospital stays and laboratory tests (including arterial partial pressure of oxygen [PaO2]) results were recorded. Results were compared with AECOPD patients having previously diagnosed and new-diagnosed type 2 diabetes. Markers associated with development of type 2 DM and the prognosis of AECOPD patients were identified.

**Results:**

Of the 196 patients enrolled in this study, the overall prevalence of T2DM was 26%. The PaO2 in the newly diagnosed T2DM group was considerably lower versus non-diabetic group. The T2DM group had a longer hospital stay and higher troponin level versus the non-diabetic group. AECOPD patients with T2DM were found to be correlated with hypertension. Age, need for assisted ventilation, increased troponin, and elevated fasting blood glucose on admission were risk factors for death in hospitalized AECOPD patients.

**Conclusions:**

AECOPD patients had a higher prevalence of T2DM than the general population; T2DM comorbidity caused lower PaO2, longer hospital stays, and increased troponin. Poor blood glucose control may increase the risk of death in AECOPD patients.

## Background

Chronic Obstructive Pulmonary Disease (COPD) exhibits persistent respiratory symptoms and airflow limitation, seriously affecting the quality of the patient’s life. Its prevalence and mortality rates are rising rapidly; the latest statistics indicate that the prevalence of COPD in people aged ≥ 40 in China is 13.7% [1] and 8.9% in America [2]. WHO estimates that by 2020 COPD may become the third leading cause of fatality in the world [3, 4], eventually leading to a huge social and economic burden.

Acute exacerbation is the leading cause of hospitalization and mortality among COPD patients. Severe exacerbation is linked to a high risk of early mortality and a median survival of only 3.6 years [5]. COPD is also often linked to several other chronic disease conditions, such as osteoporosis, cardiovascular disease, and metabolic syndrome [6–9]. Of these, D.M. is one of the most frequent and severe comorbidities. Several studies explored the incidence and prognosis of D.M. among AECOPD patients; the diagnosis of D.M. among patients hospitalized for AECOPD was about 22% in Australia [10], 18% in Switzerland [11], 22% in Taipei [12], and a high of 40% in India [13]. An investigation conducted in Finland, focusing on AECOPD among patients using inhaled beta-2-adrenergic bronchodilators and oral glucocorticoid, the prevalence of hyperglycemia was found to reach up to 82% [14]. These data were comparatively much higher than those in the general population [15, 16]. Comorbidity with hyperglycemia may cause even worse prognosis for AECOPD; the risk of hospital stay and death increases by 7–15% for each 1 mmol/L increment in blood glucose concentration [17, 18]. Hyperglycemia has been evidenced to cause a lowered quality of life, high risk of pneumonia, high risk of intensive care admission, lowered lung function, high risk of coronary heart disease (CHD), hypertension, and mortality among AECOPD patients [10, 18–20]. Nevertheless, little effort has been made to recognize the incidence and effect of T2DM among AECOPD patients, or the differences between previously diagnosed and newly diagnosed T2DM in Mainland China.

The prevalence of T2DM in hospitalized patients with AECOPD in China, factors that cause T2DM in AECOPD patients, the impact of T2DM on various indicators and prognosis of AECOPD patients, and the association with other comorbid diseases such as CHD and hypertension, with AECOPD with T2DM, have been explored. The difference between previously-diagnosed and newly diagnosed T2DM also has been discussed in this study.

## Methods

### Research subjects

This prospective observational study was conducted in patients diagnosed with AECOPD in the department of Pulmonary and Critical Care Medicine of the First Affiliated Hospital of China Medical University who were enrolled during 2011–2017. The ethics committee of the first hospital of China Medical University approved this study. This study was conducted according to the guidelines of the Declaration of Helsinki.

### Inclusion criteria and grouping

#### Diagnosis of COPD

All the patients confirmed to the clinical characteristics of COPD. Patients underwent lung function tests and exhibited a post-bronchodilator FEV1/FVC ratio of < 0.70. Patients exhibiting severe disease symptoms for whom lung function examination could not be performed at the time of hospitalization were diagnosed by a consultant in the respiratory department based on smoking history, medical history, clinical manifestations, and patients' imaging manifestations. Most of the patients have confirmed the diagnosis through pulmonary function examination at the time of discharge.

#### Diagnosis of AECOPD

AECOPD was characterized by worsening of the COPD patient’s respiratory symptoms such as cough, sputum, shortness of breath, wheezing, increased sputum volume, and purulent or purulent mucus, and can be associated with fever and other symptoms that are beyond normal day-to-day variations, acute in onset, and requires a change in regular medication [21].

#### Diagnosis of T2DM

A definite previous diagnosis of diabetes, and using a hypoglycemic agent or undergoing insulin treatment at present; fasting blood glucose ≥ 7.0 mmol/L, or random blood glucose ≥ 11.1 mmol/L or OGTT 2 h ≥ 11 in two tests conducted at 2 different times were the techniques used for diagnosing T2DM. Diagnosis of T2DM excluded the cases who exhibited normal blood glucose on admission and abnormal glucose metabolism caused by systemic application of glucocorticoid after admission and those diagnosed with T1DM.

#### Grouping

Based on the criteria mentioned above, all the patients were segregated into 3 groups: patients diagnosed with AECOPD alone were categorized into group A, patients with AECOPD and new diagnosed T2DM into group B, and patients with AECOPD and previously diagnosed T2DM were categorized under group C.

### Exclusion criteria

Patients suffering from a pulmonary embolism, pneumothorax, malignant tumor, or other serious blood system diseases were excluded from the study.

### Patient's general information and blood test

Data on the general information, clinical diagnosis, and concomitant diseases of patients such as CHD and hypertension were collected from the information gathered at admission diagnosis, auxiliary examination, and discharge diagnosis in the First Affiliated Hospital of China Medical University.

The outcomes of fasting blood glucose performed on the first morning of admission prior to treatment on that day were considered. The arterial partial pressure of oxygen (PaO2) values of all patients were in the deoxygenated state as detected on admission.

### Statistical analysis

SPSS 17.0 statistical software was used for statistical analysis, and the measured data were expressed as mean ± standard deviation (x ± SD). Analysis of normal distribution data was performed using variance. The non-normal distribution data were analyzed using the rank-sum test; the Chi-square test was used for the analysis of the correlation of CHD and hypertension with diabetes. The analyses of risk factors related to T2DM as comorbidity were performed using logistic regression analysis (The female was assigned a value of 1, and the male was assigned 0; smoking was assigned 1, and non-smoking 0; respiratory failure was assigned 1, and no respiratory failure 0, PaO2 and age were evaluated as a numerical variable). The analyses of risk factors for in-hospital deaths were also performed using logistic regression analysis (The variables were grouped and assigned values: 0.04 ng/ml troponin was used as a threshold, > 0.04 was assigned a value of 1, and < 0.04 was assigned 0; diabetes group was assigned 1 in and without diabetes 0; smoking1, while non-smoking 0; needing mechanical ventilation 1, not needing mechanical ventilation 0. Age, PaO2, and blood glucose levels were calculated as numerical variables). A value of *p* < 0.05 was considered statistically significant.

## Result

### Effect of AECOPD comorbidity with T2DM on clinical characteristics of patients

Clinical characteristics of patients of all the groups are provided in Table 1.

**Table 1:** Baseline characteristics for the total population and each group (mean ± standard deviation)

Variable	Group A	Group B	Group C	Group B + C
Total population (%)	145 (73.98)	25 (12.76)	26 (13.27)	51 (26.02)
Male gender (%)	95 (66)	16 (12.8)	14 (10.94)	30 (24)
Female gender (%)	50 (70.42)	9 (12.68)	12 (16.9)	21 (29.58)
Age	72.43 ± 8.97	72.36 ± 8.48	71.00 ± 8.99	71.65 ± 9.76
Course of disease	17.09 ± 13.51	15.04 ± 12.04	17.88 ± 12.82	16.55 ± 1.95
Hospital stays	13.58 ± 7.88	12.80 ± 8.43	19.37 ± 11.25 *^#^	16.71 ± 13.49
Death (%)	9 (6)	4 (16)	3 (12)	7 (13.73)
Comorbidities				
Hypertension (%)	39 (27)	7 (28)	15 (58)	22 (43.13)
CHD (%)	31 (21)	7 (28)	13 (50)	20 (39.22)
Laboratory values (at admission)				
Troponin (ng/ml)	0.09 ± 0.29	0.11 ± 0.39	0.37 ± 0.06 *^#^	0.25 ± 0.45
D-Dimer (µg/mL)	1.94 ± 2.11	1.61 ± 1.13	1.52 ± 0.86	1.56 ± 1.49
PaO2 (mmHg)	60.98 ± 15.05	51.66 ± 13.54**	57.11 ± 14.83	55.98 ± 14.19*
Hb (g/l)	130.28 ± 20.41	131.04 ± 13.57	128.42 ± 27.78	130.02 ± 29.51
BNP (pg/ml)	318.39 ± 625.66	414.16 ± 705.81	384.38 ± 761.74	399.47 ± 799.45

*Compared with group A *p* < 0.05; **compared with group A *p* < 0.01; ^#^compared with group B *p* < 0.05.

* CHD* Coronary heart disease

This study included 196 AECOPD patients, in which the total prevalence of AECOPD with T2DM was 26.02%, with 12.76% newly diagnosed and 13.27% previously diagnosed T2DM, respectively. The prevalence rates of female and male patients were 29.58% and 24%, respectively. Overall, 31.12% of AECOPD patients suffered hypertension, 26.02% of AECOPD patients suffered CHD, 6.63% of AECOPD patients suffered from all the 3 diseases T2DM, hypertension, and CHD. The incidences of hypertension and CHD were much higher in the previously diagnosed diabetes group than in groups without diabetes and newly diagnosed diabetes.

The PaO2 in the newly diagnosed diabetes group was recorded to be lower than that of the group without diabetes (*p* < 0.01), and also the PaO2 in the diabetic group (Group B + Group C) was observed to be lower than that in the non-diabetic group (Group A) (*p* < 0.01).

The troponin level in previously diagnosed T2DM group (Group C) was higher than the non-diabetic group (Group A) and newly diagnosed T2DM group (Group B) (*p* < 0.05).

The hospital stay period was remarkably extended in patients diagnosed earlier with diabetes compared with those without diabetes and with newly diagnosed diabetes (p < 0.05).

During this investigation, 16 patients died, with a case fatality rate of 8.16%; mortality rate without diabetes was 6%, and with diabetes was 13.73%, in which newly diagnosed diabetes was 16%, and previously diagnosed diabetes was 12%.

Correlation analysis indicated that AECOPD patients with T2DM were linked with increased troponin levels.

### Association of AECOPD comorbid diabetes with hypertension and CHD

The correlation between AECOPD with T2DM and the prevalence of hypertension and CHD were analyzed. AECOPD comorbidity with T2DM was not found to be linked to CHD. Nevertheless, in this study, AECOPD with diabetes was observed to be positively correlated with hypertension, suggesting that patients with AECOPD and T2DM were more likely to suffer from hypertension than patients with AECOPD alone (Table 2).

**Table 2:** Comorbidity with diabetes on the prevalence of hypertension of AECOPD patients

	Hypertension	Normal blood pressure	χ^2^	*p*
Group B + C	22	29	4.642	0.031
Group A	39	106		

### Reduced PaO2 positively related to comorbid T2DM

The possible risk factors such as smoking, age, gender, PaO2 reduction, and respiratory failure with the occurrence of T2DM in AECOPD patients were considered in this study. Results indicated that all the above factors could not be considered risk factors for patients previously diagnosed with T2DM. In the case of newly diagnosed patients with T2DM, reduced PaO2, and respiratory failure at admission were risk factors for diabetes (Table 3).

**Table 3:** Risk factors analysis of comorbid newly diagnosed diabetes

	B	S.E	Wals	OR	*p*
PaO2	− 0.039	0.016	6.051	0.961	0.014
Smoking	0.998	0.584	2.918	2.712	0.088
Gender	0.272	0.510	0.284	1.313	0.594
Age	− 0.008	0.027	0.081	0.992	0.775
RF	1.432	0.532	0.468	3.975	0.024

*RF* Respiratory Failure, *B* partial regression coefficient, *S.E.* standard error, *Wals* Wald test statistic, *OR* odds ratio

### Elevated fasting blood glucose at admission is one of the risk factors for in-hospital deaths

Risk factors such as old age, need for mechanical ventilation, comorbid T2DM, increased troponin levels, smoking status, and low PaO2 on the incidence of in-hospital deaths were evaluated.

After excluding other confounding factors, the factors such as old age, need for assisted ventilation, and elevated troponin posed as risk factors for death in hospitalized AECOPD patients. However, comorbid diabetes does not pose a direct risk factor for hospitalized AECOPD patients’ death (Table 4). After excluding the confounding factors in the diagnosis of diabetes, increased fasting blood glucose on admission was found to be a risk factor for the death of COPD hospitalized patients.

**Table 4:** Risk factors analysis for in-hospital death(s)

	B	S.E	Wals	OR	*p*
Age	0.139	0.05	7.567	1.149	0.006
Troponin	2.088	0.665	9.863	8.067	0.002
Smoking	− 0.041	0.772	0.003	0.960	0.957
PaO2	− 0.038	0.023	2.652	0.963	0.103
Diabetes	0.835	0.650	1.649	2.304	0.199
Ventilation	2.341	0.804	8.475	10.396	0.004

*B* partial regression coefficient, *S.E.* standard error, *Wals* Wald test statistic, *OR* odds ratio

## Discussion

In the current study, we found that the prevalence of T2DM increased in AECOPD patients, and AECOPD patients with diabetes exhibited a lower PaO2 level, increased troponin levels, and the possibility of hypertension. Lower PaO2 level also acts as a risk factor for diabetes. Risk factors for deaths during hospitalization among AECOPD patients comprise high fasting blood glucose during admission, elevated troponin, old age, and the need for assisted ventilation. Poor glycemic control influences the short-term prognosis of AECOPD patients.

AECOPD patients are at a significantly higher risk of developing T2DM than the general population [14, 22–24] and vice versa [25]. Comorbidity with T2DM has been reported to deteriorate the progression and prognosis of AECOPD patients [26].

T2DM incidence with hospitalized AECOPD patients in this study was 26.02%, which is higher than those reported in Europe and America [18, 27]. In this study, the prevalence of diabetes was 29.57% in females and 24.0% in male patients, which agree with earlier research [25]. Patients with previously diagnosed diabetes accounted for 13.27% of all surveyed AECOPD patients, whereas newly diagnosed diabetes accounted for 12.76%.

Hyperglycemia is not a well-explored therapeutic target in AECOPD. Several factors contribute to the link between AECOPD with T2DM and its adverse events [28]. First, the use of systemic and high-dose inhaled glucocorticoids in the treatment of AECOPD could likely raise the incidence of occurrence and progress of hyperglycemia [29–31], although a combined therapy of ICS and a β2-agonist may reduce this association [32]. Second, comorbidity with T2DM could raise pulmonary infection incidence, a major risk factor for AECOPD [33]. Third, hypoxia [34, 35], acidosis [36], systemic inflammatory response [28], and stress [14, 33, 37] among AECOPD patients could trigger the glucose-elevating hormones and lead to the occurrence of hyperglycemia. Fourth, hyperglycemia also could have adverse effects on the lungs because it is likely to stimulate the glycosylation of connective tissues, reduce pulmonary elastic recoil, cause weakness and/or inflammation in respiratory muscles, and susceptibility to bacterial infection, which increase the risk of AECOPD [38–41]. Fifth, chronic hyperglycemia also can induce systemic vascular and nerve damage on lung histopathology, and lung function changes consistently with the systemic microvascular disease [42]. Last, hypoglycemia raises the aggregation of blood platelets and fibrinogen formation, which may speed up the vascular damage in the lung and cause a higher risk for in-hospital complications and longer hospitalizations [43, 44].

In this study, patients with T2DM exhibit a lower PaO2, whereas patients with newly diagnosed T2DM show even lower PaO2 than patients with preexisting T2DM. Moreover, regression analysis indicated that a lower PaO2 and respiratory failure were risk factors for AECOPD patients with newly developed T2DM. It is incomprehensive to identify the reasons for the damage to pulmonary dispersion function caused by diabetes, as the effect of high blood glucose on small airway and the effect of pulmonary dispersion functions progress over time. The correlation between hypoxemia and abnormal glucose tolerance has been explored [9], and oxygen therapy was found to improve the glucose tolerance of AECOPD patients significantly. Although the normal PaO2 levels could not be restored, there was a remarkable difference compared with that before the therapy. However, Oltmanns et al. found that reducing blood oxygen saturation to 75% of its original level in healthy subjects within 30 min added abnormal glucose tolerance. The mediating cause of abnormal glucose tolerance is attributable to elevated levels of adrenaline [35]. In an earlier investigation, increased adrenalin secretion has been reported to likely cause an increase in serum adrenalin levels among patients with AECOPD acute episode [45]. Hypoxemia and the rise in PaO2 caused by oxygen therapy are likely to influence the concentration of catecholamines in blood, which can rapidly influence the sensitivity of peripheral tissues to insulin. It could be assumed that the reason for the prevalence of diabetes in this study to be higher than that reported in the above-mentioned investigation is the stress caused by AECOPD, causing a high catecholamine secretion, thus influencing glucose tolerance. Nevertheless, the difference in PaO2 values between patients with newly diagnosed T2DM and patients with previously diagnosed T2DM may cause abnormal glucose tolerance and even the diagnosis of diabetes, rather than vice versa. The 2 factors may also be mutually promoting, and further investigations are obligatory.

Troponin level in T2DM group was found to be higher than that in the non-diabetic group as per the statistical outcomes of this study, and in agreement with the existing research results, troponin was found to be one of the risk factors for the death of hospitalized patients [46]. Thus, T2DM may indirectly influence the prognosis of hospitalized AECOPD patients, of course, as observed in this investigation. Age and the level of blood glucose control also influence the incidence of T2DM in AECOPD patients, and in the end, influence the prognosis of patients, which is more worthy of attention. Only 7 of the 27 patients with elevated troponin were diagnosed with CHD, and 20 had no history or manifestation of CHD. Troponin elevation is the most sensitive and specific biochemical indicator of ischemic myocardial injury. However, non-ischemic myocardial injury-related pathological conditions such as sepsis, pulmonary embolism, viral myocarditis, and renal failure are also likely to occur, and as one of the critical factors influencing long-term prognosis [47]. The autonomic nervous system dysfunction causes overactivation of the sympathetic nerve and an increase in catecholamine effect in cardiomyocytes. Injury to myocardial cells resulting from trauma or inflammation is part of the possible mechanism, and hypoxemia may also cause myocardial injury. Moreover, among AECOPD patients, these are most likely occurrences.

In this study, AECOPD with T2DM was positively correlated with hypertension, which means that when AECOPD patients suffer from T2DM, patients are more likely to have hypertension, which deserves more attention. It was not evidenced that T2DM was a risk factor for CHD in AECOPD patients because the association between T2DM and CHD was not statistically significant in this study, which was not in agreement with several earlier reports in T2DM patients. We attribute this to inadequate clinical diagnosis of CHD or sample size, and also COPD is an independent risk factor for CHD because they share some same systemic inflammatory mechanisms [48], which may weaken the connection between D.M. and CHD in AECOPD patients in this study.

Poor control of diabetes has been reported to relate to a higher prevalence of AECOPD, and poor control of diabetes of hospitalized patients can prolong the length of hospital stay and increase the probability of complications due to comorbidities and in-hospital mortality, including cardiovascular deaths [3].

Aged, need for assisted ventilation, and elevated troponin were risk factors for death in hospitalized AECOPD patients. These results were consistent with findings of earlier studies [18, 49]; however, comorbid T2DM does not pose a direct risk factor for hospitalized AECOPD patients’ death. As fasting blood glucose higher than 11.0 mmol/l at admission has been evidenced to pose a risk factor for hospitalized deaths of AECOPD patients [18]. A simple analysis of whether a patient has diabetes or not may inadequate to evaluate patients' blood glucose levels since even patients with T2DM may have different blood glucose control conditions. Therefore, we calculated the correlation between the death of hospitalized patients and fasting blood glucose. After excluding the confounding factors in the diagnosis of diabetes, increased fasting blood glucose on admission was found to be a risk factor for the death of COPD hospitalized patients.

AECOPD patients are at a higher risk of T2DM than the general population, which may increase these AECOPD patients' short-term mortality, especially in the newly diagnosed T2DM group. We found that AECOPD patients with newly diagnosed T2DM suffer from lower PaO2. As lower PaO2 is also an independent risk factor for T2DM, PaO2 levels should be carefully observed among AECOPD patients, and oxygen therapy is necessary for patients with AECOPD combined with hypoxia, which is likely to reduce the incidence of T2DM. When AECOPD patients have T2DM as comorbidity, they were more likely to suffer from hypertension than CHD; in that case, we should pay more attention to their blood pressure. Old age, need for assisted ventilation, elevated troponin, and increasing fasting blood glucose increase the short-term mortality in hospitalized AECOPD patients; in that case, AECOPD patients with abnormal blood glucose should be prioritized; early detection and timely control and oxygen therapy are some of the effective measures that are eventually beneficial to patients with diabetes in terms of improving their quality of life.

## Conclusion

The prevalence of diabetes increased in AECOPD patients, and AECOPD patients with diabetes exhibited a lower PaO2 level, increased troponin levels, and the possibility of hypertension. Lower PaO2 level also acts as a risk factor for diabetes. Risk factors for deaths during hospitalization among AECOPD patients comprise high fasting blood glucose during admission, elevated troponin, old age, and the need for assisted ventilation. Poor glycemic control influences the short-term prognosis of AECOPD patients. AECOPD patients with diabetes and abnormal blood glucose should be prioritized; early detection and timely control as well as oxygen therapy are some of the effective measures that are eventually beneficial to patients with diabetes in terms of improving their quality of life.

## Data Availability

The datasets used and/or analyzed during the current study are available from the corresponding author on reasonable request.

## Abbreviations

T2DM: Type 2 diabetes mellitus
T1DM: Type 1 diabetes mellitus
AECOPD: Acute exacerbation of COPD
COPD: Chronic obstructive pulmonary disease
PaO2: Arterial partial pressure of oxygen
D.M.: Diabetes mellitus
CHD: Coronary heart disease
OGTT: Oral glucose tolerance test
FEV1: Forced expiratory volume in the first second
FVC: Forced vital capacity
